# Prevalence and Incidence of Non-alcohol Fatty Liver Disease in Chronic Hepatitis B Population in Southeast China: A Community-Based Study

**DOI:** 10.3389/fmed.2021.683872

**Published:** 2021-07-19

**Authors:** Yang Zheng, Kaijin Xu, Haiyang Hu, Mohamed S. Draz, Wei Wu, Lanjuan Li

**Affiliations:** ^1^State Key Laboratory for Diagnosis and Treatment of Infectious Diseases, National Clinical Research Centre for Infectious Diseases, Collaborative Innovation Centre for Diagnosis and Treatment of Infectious Diseases, The First Affiliated Hospital, College of Medicine, Zhejiang University, Hangzhou, China; ^2^Department of General Practice, The First Affiliated Hospital, College of Medicine, Zhejiang University, Hangzhou, China; ^3^Department of Medicine, Case Western Reserve University School of Medicine, Cleveland, OH, United States

**Keywords:** non-alcohol fatty liver disease, prevalence, incidence, chronic hepatitis B infection, metabolic factor

## Abstract

**Background:** To investigate the prevalence and incidence of non-alcohol fatty liver disease (NAFLD) in a community-based chronic hepatitis B (CHB) population from Southeast China and evaluate the association between NAFLD and metabolic factors, viral factors, and underlying chronic diseases.

**Methods:** CHB patients were recruited in 2012 and followed up from 2017 to 2019 in Zhejiang, China. NAFLD prevalence of the last visit and NAFLD incidence were calculated. Potential risk factors, including metabolic and viral factors, were also evaluated using Logistic or Cox regression models.

**Results:** NAFLD prevalence of the last visit in 2019 was estimated at 26.76%. Waist circumference, body mass index (BMI), triglyceride (TG), low-density lipoprotein (LDL), and diabetes mellitus (DM) were found as associated factors. In subgroups analysis, HBV infection types were also identified as a risk factor in the non-diabetic population. HBeAg-negative hepatitis and immunotolerant had lower NAFLD prevalence than past CHB infection. NAFLD incidence was estimated at 22.63/1,000 person-years after 1,634.74 person-years of follow-up. Waist circumference, TG, LDL, and alkaline phosphatase (ALP) were identified as associated factors.

**Conclusion:** The NAFLD prevalence and incidence in our study were slightly lower than previous reports from East Asia. Health education and healthy living habits were extremely important in reducing the NAFLD burden. Metabolic factors, history of DM, and viral factors were associated with NAFLD in CHB patients.

## Significance Statement

This article investigates an updated prevalence and incidence of NAFLD in a community CHB population from Southeast China. It comprehensively evaluates the association between NAFLD and viral factors, metabolic factors, and underlying chronic diseases. We find that NAFLD prevalence in 2019 was 26.76%, NAFLD incidence during 2017–2019 was 22.63/1,000 person-years, both of which were slightly lower than previous reports in Asia. Health education and healthy living habits were critical in reducing the NAFLD burden. Metabolic factors and diabetes mellitus (DM) were identified as associated with NAFLD. Factors related to the virus were also connected with NAFLD in the non-diabetic population.

## Introduction

Over the last decade, the prevalence and incidence of overweight and associated diseases, such as non-alcohol fatty liver disease (NAFLD), have increased dramatically in China (1, 2). It is now one of the most common liver diseases in China—an estimate of 29.2% of the general Chinese population is reported to suffer NAFLD in 2018 (2), and this figure is expected to continue to increase in the future rapidly (3).

Among the chronic hepatitis B (CHB) infected population, the co-morbidity of NAFLD was also common. Although CHB patients tended to have less NAFLD prevalence rate than the general population, as suggested by prior findings (4), the concurrent NAFLD in CHB is still an increasingly alarming problem. This is because of the high risk of developing liver complications, including fibrosis, cirrhosis, and hepatocellular carcinoma (HCC) in patients with CHB, and NAFLD (5, 6). Several prior studies had evaluated the regional epidemiology and risk factors of NAFLD in CHB patients in China. A hospital-based study with 14,452 patients showed that the prevalence of NAFLD ranged from 29.9% to 35.8% in CHB, which was lower than past-infection and general population (4). Another study in the South of China reported a lower prevalence of NAFLD (24.1%) in hospitalized CHB patients, with central obesity, hypertension, hypertriglyceridemia, non-cirrhosis were risk factors (7). A cohort study in East China that followed up 2,393 CHB patients for 3 years demonstrated a NAFLD incidence rate of 63.89/1,000 with influencing factors of obesity and type 2 DM (8). However, a comprehensive association between viral factors, metabolic factors, underlying chronic diseases, and the incidence of NAFLD was rarely investigated in the previous; meanwhile, little investigation has reported NAFLD's prevalence or incidence rate in CHB population (9–11).

Therefore, in this study, we aimed to investigate an updated condition: (1) to track recent changes in the prevalence and incidence of NAFLD in a community CHB population from Southeast China, and (2) to comprehensively evaluate the association between NAFLD and viral factors, metabolic factors, and underlying chronic diseases—in a broader population.

## Materials and Methods

### Study Population Enrollment and Follow-Up

A total of 1,762 CHB patients residing in Zhejiang, Southeast China, who had a previous examination community-based collaborative innovation (CCI) project for the prevention and control of hepatitis B in 2012 were included this study (12). In brief, a multistage stratified random cluster sampling was performed in the prior hepatitis B virus (HBV) epidemiological study. We considered geographic characteristics and chose several cities as selected locations. Towns in each city were ranked into three tiers according to economic levels, with one town randomly selected at each tier. Three villages were eventually selected from different town tiers. All residents in each selected village were recruited (13). Our study included all chronic HBV-infected residents from three villages (Qianqing, Qixian, Anchang) of Shaoxing City, Zhejiang. After excluding heavy alcohol intake (ethanol dosage >20 g/d in male, >10 g/d in female) and inability to cooperate, 1,125 individuals who met the criteria of HBsAg positive for 6 months of 2012 were enrolled. Follow-up started in March 2017 and ended in December 2019 with an interval of 1 year. Demographic data and clinical information, including age, gender, body mass index (BMI), waist circumference, blood pressure, and history of DM were collected from the electronic database of the local medical insurance system. During each visit, laboratory tests including complete blood cell count, liver function, renal function, serum lipid [triglyceride (TG), total cholesterol (TC), low-density lipoprotein (LDL), high-density lipoprotein (HDL)], fasting glucose, alpha-fetoprotein, HBV serology (HBsAg, anti-HBs, HBeAg, anti-HBe, anti-HBc), and HBV DNA were checked. Continuous serum lipid variables, waist circumference, and BMI were transformed into categorical variables as described in Supplementary S1. Abdominal ultrasound was first evaluated in 2017 and followed up annually by community healthcare teams.

This study was approved by the Ethics Committee of the First Affiliated Hospital, College of Medicine, Zhejiang University, following Helsinki's Declaration. Informed consent was acquired from all participants.

### Laboratory Testing and Diagnosis Criteria

Blood samples were kept in cool containers and delivered to Adicon Clinical Laboratories (Hangzhou, China) for processing and testing. ELISA kits (Acon Biotech Co., Hangzhou, China) were used for HBV serology. A real-time fluorescent PCR system (7300; Applied Biosystems, Inc., Carlsbad, CA, USA) was used for HBV DNA levels. Architect C8000 automated biochemical analyzer (Abbott Laboratories, Abbott Park, IL, USA) was used for liver function, renal function, serum fasting glucose, and serum lipids.

Chronic HBV infection patients were classified as immunotolerant patients, inactive carriers, HBeAg-positive hepatitis, HBeAg-negative hepatitis, and past infection (diagnose criteria described in Supplementary S2). NAFLD was diagnosed with abdominal ultrasound with features of increased echogenicity of the liver parenchyma appearing brighter than the kidney's cortex, intrahepatic vessels blurring, and deep attenuation (14).

### Statistical Analysis

**Figure 1:**
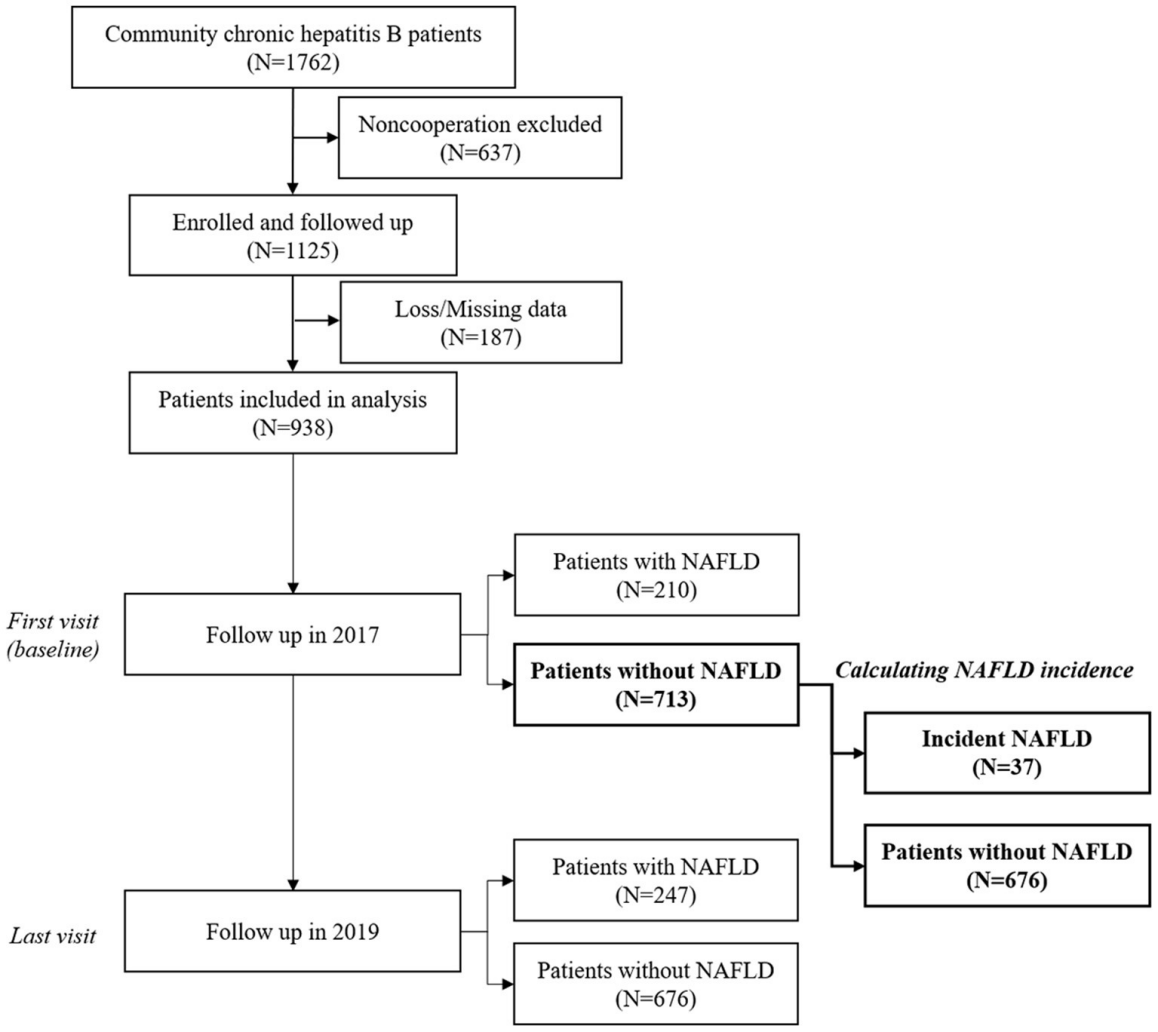
Flow chart and timeline of patient selection and follow-up. NAFLD, non-alcohol fatty liver disease.

In our work, the data of the last visit in 2019 was used to reveal the most updated NAFLD prevalence. Those who had no NAFLD at the first visit in 2017 were further recruited for calculating the incidence of NAFLD (Figure 1). Incidence of NAFLD was calculated using the incident NAFLD cases who did not have NAFLD on initial ultrasound but developed on the subsequent visits divided by summed person-year of follow-up. Duration of follow-up was defined as the date of baseline visit to the date of last visit or NAFLD development. Subgroup analysis was performed according to DM status.

Continuous variables data were expressed as median (IQR), categorical variable data were as a percentage. Differences in demographic and clinical characteristics were calculated using *t*-test or Mann-Whitney *U*-test for continuous variables and the chi-square test or Fisher's exact-test for categorical variables. In exploring the influencing factors of NAFLD prevalence, odds ratios (ORs) were calculated using logistic regression models. In examining the influencing factors of NAFLD incidence, hazard ratios (HRs) were calculated using Cox regression models. Variables with *p* < 0.10 in univariate analysis were included in a stepwise logistic or Cox regression model. SPSS version 25.0 (Chicago, IL, USA) was used for data analysis. *p* < 0.05 was considered statistically significant.

## Results

### Characteristics of Participants at Enrollment

A total of 1,125 individuals were enrolled at baseline, while 202 lost to follow-up or presented missing data. Finally, there were altogether 923 individuals included in the analysis. Of them, the median age in 2017 was 61.0 (IQR 54.0–68.0), 442 (47.9%) were male, and 55 (5.96%) had DM. According to baseline HBV serology, HBV DNA and liver function (Supplementary S2), there were 55 (5.9%) immunotolerant patients, 734 (78.3%) inactive carrier, 28 (3.0%) HBeAg-positive hepatitis, 80 (8.5%) HBeAg-negative hepatitis, and 41 (4.4%) past infection. There were 13 (1.4%) cases of cirrhosis.

### Prevalence and Associated Factors of NAFLD

In 2019, there were 247 (26.76%) NAFLD among 923 CHB patients, whose characteristics are shown in Supplementary Table 1. As shown in Table 1, the presence of NAFLD was associated with waist circumference, BMI, DM, blood pressure, ALT, BUN, TG, LDL, and HDL in univariate analysis. In multivariate analysis, waist circumference (OR = 1.66), BMI (overweight: OR = 2.53; obese: OR = 7.22), DM (OR = 2.10), TG (intermediate: OR = 3.47; high: OR = 4.98), and LDL (intermediate: OR = 2.29; high: OR = 3.85) were independently associated with the risk of NAFLD in CHB patients.

**Table 1:** Risk factors associated with the prevalence of NAFLD.

**Variables**	**NAFLD (*n* = 247)**	**Non-NAFLD (*n* = 676)**	**Univariate logistic regression**		**Multivariate logistic regression**	
			**OR (95% CI)**	***p***	**OR (95% CI)**	***p***
**Demographics and clinical characteristics**						
**Age (in 2019)**	63.0 (56.0–69.0)	64.0 (55.0–70.0)	1.00 (0.99–1.02)	0.645		
**Gender**						
Male	112 (45.3%)	330 (48.8%)	1 (ref)			
Female	135 (54.7%)	346 (51.2%)	1.15 (0.86–1.54)	0.350		
**Waist**	90.0 (84.0–95.0)	81.0 (75.0–87.0)				
Normal	84 (36.2%)	472 (74.7%)	1 (ref)		1 (ref)	
Obesity	148 (63.8%)	160 (25.3%)	5.20 (3.77–7.17)	<0.001	1.66 (1.06–2.59)	0.026
**BMI**	26.73 ± 3.01	23.37 ± 2.93				
Normal	43 (18.5%)	367 (57.8%)	1 (ref)		1 (ref)	
Overweight	85 (36.6%)	196 (30.9%)	3.70 (2.47–5.55)	<0.001	2.53 (1.56–4.08)	<0.001
Obese	104 (44.8%)	72 (11.3%)	12.33 (7.97–19.07)	<0.001	7.22 (4.04–12.91)	<0.001
**DM**						
–	220 (90.1%)	648 (95.9%)	1 (ref)		1 (ref)	
+	27 (10.9%)	28 (4.1%)	2.84 (1.64–4.93)	<0.001	2.10 (1.09–4.07)	0.028
**SBP, mmHg**	143 (133–156)	136 (125–151)	1.02 (1.01–1.03)	<0.001		
**DBP, mmHg**	84 (77–91.8)	81 (74–89)	1.02 (1.01–1.04)	0.001		
**Laboratory findings**						
**ALT, U/L**	26.0 (20.0–40.8)	21.0 (16.2–30.0)	1.01 (1.00–1.02)	0.004		
<50	210	623	1 (ref)			
≥50	37	53	1.98 (1.26–3.09)	0.001		
**BUN, mmol/L**	5.09 (4.28–6.06)	5.47 (4.50–6.45)	0.87 (0.78–0.97)	0.01		
<8	219(88.7%)	546 (80.8%)	1 (ref)			
≥8	8 (3.2%)	41 (6.2%)	0.49 (0.22–1.05)	0.068		
**TG**	1.85 (1.19–2.50)	1.03 (0.77–1.48)				
Optimal	100 (42.9%)	480 (79.9%)	1 (ref)		1 (ref)	
Intermediate	61 (26.2%)	74 (12.3%)	3.96 (2.65–5.91)	<0.001	3.47 (2.19–5.49)	<0.001
High	72 (30.9%)	47 (7.8%)	7.35 (4.80–11.26)	<0.001	4.98 (3.04–8.14)	<0.001
**LDL**	2.73 (2.23–3.36)	2.38 (1.98–2.82)				
Optimal	175 (75.1%)	545 (90.8%)	1 (ref)		1 (ref)	
Intermediate	46 (19.7%)	51 (8.5%)	2.81 (1.82–4.33)	<0.001	2.29 (1.37–3.83)	0.002
High	12 (5.2%)	4 (0.7%)	9.34 (2.98–29.34)	<0.001	3.85 (0.95–15.57)	0.059
**HDL**	1.34 (1.16–1.58)	1.46 (1.25–1.70)				
Optimal	63 (27.0%)	227 (37.8%)	1 (ref)			
Intermediate	146 (62.7%)	335 (55.8%)	1.57 (1.12–2.21)	0.009		
High	24 (10.3%)	38 (6.3%)	2.28 (1.27–4.07)	0.006		

*Data are median (IQR) or median ± S.D for continuous variables, n (%) for categorical variables. Age is measured in the year of 2019*.

*NAFLD, non-alcohol fatty liver disease; BMI, body mass index; DM, diabetes mellitus; SBP, systolic blood pressure; DBP, diastolic blood pressure; ALT, alanine transaminase; BUN, blood urine nitrogen; TG, triglycerides; LDL, low density lipoprotein; HDL, high density lipoprotein*.

### NAFLD Prevalence of Subgroup Population Stratified by Diabetes Mellitus

Supplementary Table 2 showed the characteristics of diabetes and non-diabetes groups. Compared with the non-diabetic group, NAFLD was much more common in diabetic (49.1 vs. 25.3%, *p* < 0.001). The diabetic population also had higher TG, TC, and a different proportion of HBV type relative to the non-diabetic population (*p* < 0.05). The age, gender, waist circumference, BMI, LDL, and HDL were not significantly different between these subgroups.

In the non-diabetic subgroup, univariate analysis revealed the presence of NAFLD was associated with ALT, waist circumference, BMI, TG, LDL, HDL, and HBV type. In multivariate analysis, waist circumference (OR = 1.69), BMI (overweight: OR = 2.53; obese: OR = 7.51), TG (intermediate: OR = 3.35; high: OR = 4.53), and LDL (intermediate: OR = 2.26; high: OR = 4.21) were identified independent factors. Additionally, HBV type was also found an association, with lower NAFLD prevalence in HBeAg-negative hepatitis (OR = 0.32) and immunotolerant (OR = 0.22) compared to past infection.

In the diabetic subgroup, univariate analysis revealed the presence of NAFLD was associated with gender, waist circumference, BMI, and TG. In multivariate analysis, female (OR = 8.22), waist circumference (OR = 4.39), and TG (intermediate: OR = 6.04; high: OR = 16.49) were identified independent factors (Table 2).

**Table 2:** Effects of risk factors for NAFLD prevalence in diabetic and non-diabetic patients.

	**Non-DM (*****n*** **=** **868)**				**DM (*****n*** **=** **55)**			
	**Crude OR**	***p***	**Adjust OR**	***p***	**Crude OR**	***p***	**Adjust OR**	***p***
**Age**	1.00 (0.99–1.02)	0.617			0.96 (0.89–1.04)	0.332		
**Gender**								
Male	1 (ref)				1 (ref)		1 (ref)	
Female	1.04 (0.77–1.42)	0.790			2.86 (0.92–8.89)	0.070	8.22 (1.24–54.60)	0.029
**ALT, U/L**								
<50	1 (ref)				1 (ref)			
≥50	2.10 (1.31–3.38)	0.002			0.80 (0.19–3.36)	0.761		
**Waist**								
Normal	1 (ref)		1 (ref)		1 (ref)		1 (ref)	
Obesity	5.36 (3.82–7.52)	<0.001	1.69 (1.06–2.69)	0.027	3.06 (1.02–9.18)	0.046	4.39 (1.16–16.57)	0.029
**BMI**								
Normal	1 (ref)		1 (ref)		1 (ref)			
Overweight	3.85 (2.50–5.92)	<0.001	2.53 (1.54–4.17)	<0.001	2.81 (0.73–10.77)	0.131		
Obese	13.06 (8.23–20.74)	<0.001	7.51 (4.09–13.79)	<0.001	6.00 (1.47–24.55)	0.013		
**TG**								
Optimal	1 (ref)		1 (ref)		1 (ref)		1 (ref)	
Intermediate	3.69 (2.41–5.64)	<0.001	3.35 (2.07–5.44)	<0.001	5.25 (1.37–20.20)	0.016	6.04 (1.40–26.01)	0.016
High	6.99 (4.48–10.88)	<0.001	4.53 (2.72–7.53)	<0.001	11.81 (2.08–66.97)	0.005	16.49 (2.51–108.40)	0.004
**LDL**								
Optimal	1 (ref)		1 (ref)		1 (ref)			
Intermediate	2.84 (1.80–4.49)	<0.001	2.26 (1.32–3.87)	0.003	1.90 (0.47–7.69)	0.371		
High	8.35 (2.58–26.99)	<0.001	4.21 (1.02–17.28)	0.046	-			
**HDL**								
Optimal	1 (ref)				1 (ref)			
Intermediate	1.70 (1.19–2.44)	0.004			0.57 (0.17–1.91)	0.365		
Low	2.26 (1.22–4.22)	0.01			1.56 (0.22–11.09)	0.659		
**HBV type**								
Past infection	1 (ref)		1 (ref)					
e– hepatitis	0.66 (0.29–1.52)	0.33	0.32 (0.11–0.97)	0.044				
e+ hepatitis	0.52 (0.16–1.69)	0.274	0.59 (0.14–2.61)	0.49	-			
Inactive carrier	0.65 (0.33–1.27)	0.203	0.57 (0.24–1.32)	0.188	-			
Immunotolerant	0.27 (0.10–0.76)	0.013	0.22 (0.06–0.85)	0.028	-			

*Age, gender, and other variables that are statistically significant in multivariable Logistic regression are presented with adjust OR and p-value. Crude/Adjust OR is presented as point estimate (lower limit-upper limit of 95% confidential interval)*.

*NAFLD, non-alcohol fatty liver disease; DM, diabetes mellitus; OR, odds ratio; ALT, Alanine transaminase; BMI, body mass index; TG, triglycerides; LDL, low density lipoprotein; HDL, high density lipoprotein*.

### Incidence and Associated Factors of NAFLD

A total of 713 eligible CHB patients with negative results of baseline abdominal ultrasound in 2017 were recruited. After a median of 30.63 months (IQR 19.84–32.60 months) of follow-up, 37 (5.08%) developed new NAFLD. Over 1,634.74 person-years, the incidence rate of NAFLD was 22.63/1,000 person-years. The characteristics of incident NAFLD and non-NAFLD groups are shown in Supplementary Table 3.

In Table 3, the univariate analysis demonstrated that waist circumference, BMI, DM, ALP, TG, and LDL were associated with the development of new NAFLD. In the multivariate Cox regression model, obesity waist circumference (HR = 2.47, 95% CI 1.25–4.88), ALP ≥ 125 U/L (HR = 0.25, 95%CI 0.09–0.73), TG (intermediate: HR = 2.76, 95%CI 1.23–6.17; high: HR = 3.00, 95%CI 1.28–7.04), and LDL (intermediate: HR = 2.96, 95%CI 1.17–7.52; high: HR = 8.26, 95%CI 2.57–26.50) were independently related to incident NAFLD.

**Table 3:** Risk factors associated with the incidence of NAFLD.

**Variables**	**Incidence (*n* = 37)**	**Negative(*n* = 676)**	**Univariate cox regression**		**Multivariate cox regression**	
			**HR (95% CI)**	***p***	**HR (95% CI)**	***p***
**Demographics and clinical characteristics**						
**Age (in 2017)**	61.0 (55.5–67.0)	62.0 (53.0–68.0)	1.00 (0.97–1.04)	0.977		
**Gender**						
Male	17 (45.9%)	330 (48.8%)	1 (ref)			
Female	20 (54.1%)	346 (51.2%)	1.19 (0.62–2.28)	0.594		
**Waist**	88.0 (83.0–93.5)	81.0 (75.0–87.0)	1.11 (1.06–1.15)	<0.001	1.09 (1.04–1.13)	<0.001
Normal	17 (45.9%)	472 (74.7%)	1 (ref)		1 (ref)	
Obesity	20 (54.1%)	160 (25.3%)	3.11 (1.63–5.94)	<0.001	2.47 (1.25–4.88)	0.009
**BMI**	25.56 ± 2.46	23.40 ± 2.96	1.25 (1.13–1.38)	<0.001		
Normal	11 (29.7%)	367 (57.8%)	1 (ref)			
Overweight	11 (29.7%)	196 (30.9%)	1.71 (0.74–3.95)	0.207		
Obese	15 (40.5%)	72 (11.3%)	6.10 (2.80–13.28)	<0.001		
**DM**						
–	33 (89.2%)	648 (95.9%)	1 (ref)			
+	4 (10.8%)	28 (4.1%)	2.49 (0.89–7.02)	0.085		
**HBV type**						
Past infection	3 (8.1%)	27 (4.0%)	1 (ref)			
e– hepatitis	0 (0.0%)	58 (8.6%)	-	0.974		
e+ hepatitis	3 (8.1%)	20 (3.0%)	1.42 (0.29–7.05)	0.667		
Inactive carrier	31 (83.8%)	523 (77.4%)	0.55 (0.17–1.79)	0.319		
Immunotolerant	0 (0.0%)	48 (7.1%)	-	0.976		
**Laboratory findings**						
**ALP, U/L**	89.0 (73.5–107.5)	98.0 (78.0–120.0)	0.99 (0.98–1.00)	0.034		
<125	33 (89.2%)	536 (79.3%)	1 (ref)		1 (ref)	
≥125	4 (10.8%)	140 (20.7%)	0.38 (0.13–1.07)	0.068	0.25 (0.09–0.73)	0.011
**TG**	1.70 (1.11–2.12)	1.03 (0.77–1.48)	1.42 (1.18–1.70)	<0.001	1.47 (1.15–1.88)	0.002
Optimal	18 (48.6%)	475 (80.4%)	1 (ref)		1 (ref)	
Intermediate	11 (29.7%)	72 (12.2%)	3.93 (1.85–8.35)	<0.001	2.76 (1.23–6.17)	0.013
High	8 (21.6%)	44 (7.4%)	3.97 (1.72–9.13)	0.001	3.00 (1.28–7.04)	0.012
**LDL**	2.94 (2.10–3.43)	2.38 (1.98–2.82)	2.37 (1.58–3.55)	<0.001	1.92 (1.24–2.98)	0.004
Optimal	27 (73.0%)	543 (91.1%)	1 (ref)		1 (ref)	
Intermediate	6 (16.2%)	49 (8.2%)	2.86 (1.17–6.99)	0.021	2.96 (1.17–7.52)	0.022
High	4 (10.8%)	4 (0.7%)	12.40 (4.30–35.72)	<0.001	8.26 (2.57–26.50)	<0.001

*Data are median (IQR) or median ± S.D for continuous variables, n (%) for categorical variables. Age is measured in the year of 2017*.

*NAFLD, non-alcohol fatty liver disease; BMI, body mass index; DM, diabetes mellitus; ALP, alkaline phosphatase; TG, triglycerides; LDL, low density lipoprotein*.

### NAFLD Incidence of Subgroup Population Stratified by Diabetes Mellitus

Supplementary Table 4 shows the characteristics of diabetic and non-diabetic groups. No significant difference was found in demographic and lab results between the two subgroups. In non-diabetic, univariate results revealed ALP, waist circumference, BMI, TG, and LDL were associated with incident NAFLD. The multivariate Cox regression model demonstrated ALP ≥ 125 U/L (HR = 0.20, 95%CI 0.06–0.69), waist circumference (HR = 2.31, 95%CI 1.14–4.71), TG (intermediate: HR = 3.12, 95%CI 1.35–7.21; high: HR = 3.61, 95%CI 1.44–9.04), and LDL (high: HR = 7.03, 95%CI 1.90–25.97) were independent factors. In the diabetic subgroup, the multivariate Cox regression model didn't indicate any statistically significant variables (Table 4).

**Table 4:** Effects of risk factors for NAFLD incidence in diabetic and non-diabetic patients.

	**Non-DM (*****n*** **=** **681)**				**DM (*****n*** **=** **32)**			
	**cHR**	***p***	**aHR**	***p***	**cHR**	***p***	**aHR**	***p***
**Age**	1.01 (0.97–1.05)	0.572			0.84 (0.71–0.99)	0.039		
**Gender**								
Male	1 (ref)				1 (ref)			
Female	1.07 (0.54–2.13)	0.839			3.45 (0.36–33.44)	0.285		
**ALP, U/L**						0.935		
<125	1 (ref)		1 (ref)		1 (ref)			
≥125	0.32 (0.10–1.03)	0.057	0.20 (0.06–0.69)	0.011	0.91 (0.09–9.05)			
**Waist**	1.11 (1.07–1.16)	<0.001	1.09 (1.04–1.13)	<0.001	1.05 (0.931–1.18)	0.434		
Normal	1 (ref)		1 (ref)					
Obesity	2.87 (1.45–5.68)	0.002	2.31 (1.14–4.71)	0.021	6.33 (0.63–63.29)	0.116		
**BMI**	1.25 (1.11–1.39)	<0.001			1.20 (0.96–1.50)	0.113		
Normal	1 (ref)				1 (ref)			
Overweight	1.73 (0.72–4.15)	0.222			1.57 (0.10–25.08)	0.751		
Obese	5.90 (2.59–13.46)	<0.001			10.84 (0.69–171.33)	0.091		
**TG**	1.42 (1.17–1.71)	<0.001			1.89 (0.58–6.23)	0.294		
Optimal	1 (ref)		1 (ref)		1 (ref)			
Intermediate	4.15 (1.88–9.19)	<0.001	3.12 (1.35–7.21)	0.008	2.26 (0.19–27.52)	0.522		
High	3.97 (1.63–9.66)	0.002	3.61 (1.44–9.04)	0.006	3.28 (0.28–38.03)	0.342		
**LDL**	2.31 (1.49–3.58)	<0.001			2.13 (0.75–6.05)	0.155		
Optimal	1 (ref)		1 (ref)		1 (ref)			
Intermediate	2.62 (0.99–6.89)	0.051	1.97 (0.73–5.26)	0.449	5.96 (0.37–96.46)	0.209		
High	10.36 (3.11–34.55)	<0.001	7.03 (1.90–25.97)	0.003	19.11 (1.19–306.09)	0.037		
**HBV Type**		0.668				0.925		
Past infection	1 (ref)				1 (ref)			
e– hepatitis	-				-			
e+ hepatitis	1.05 (0.18–6.31)	0.955			-			
Inactive carrier	0.48 (0.15–1.59)	0.233			-			
Immunotolerant	-				-			

*Age, gender, and other variables that are statistically significant in multivariable Cox regression are presented with adjust HR and p-value. Crude/Adjust HR is presented as point estimate (lower limit-upper limit of 95% confidential interval). Age is measured at baseline in the year of 2017*.

*NAFLD, non-alcohol fatty liver disease; DM, diabetes mellitus; HR, hazard ratio; ALP, alkaline phosphatase; BMI, body mass index; TG, triglycerides; LDL, low-density lipoprotein; HBV, hepatitis B virus*.

## Discussion

This community-based study provides a comprehensive overview of the NAFLD prevalence and incidence in the CHB population. The epidemiology of NAFLD in CHB patients from China was rarely reported before or not updated. We indicated that both rates of prevalence and incidence in our results were slightly lower than those reported in previous studies. Multiple metabolic factors such as waist circumference, BMI, TG, LDL, and underlying chronic health conditions such as DM were connected with NAFLD. Of particular note, we further revealed an association between a viral factor (i.e., HBV infection types) and NAFLD prevalence in the non-diabetic subgroup, with HBeAg-negative hepatitis and immunotolerant patients, who had lower NAFLD than past infection patients.

The estimated prevalence and incidence rates of NAFLD in our study were slightly lower than the results of other epidemiological studies in East Asia evaluated by ultrasound. For NAFLD prevalence, cross-sectional studies in 2013 from Taiwan revealed that the prevalence of NAFLD was 38.9–44.5% (15, 16). Another Southeast Chinese study in 2019 estimated that the NAFLD prevalence was 29.9 and 35.8% for current and past HBV infected groups, respectively (4). For NAFLD incidence, a Korean cohort study followed up 484,736.1 person-years and identified the incidence of 20,200 NAFLD cases (till 2014) in a chronic HBV-infected population, which yielded an incidence of 41.67/1,000 person-year (17). Another recent cohort from East China reported an incidence rate of 63.89/1,000 person-year after 2 years to follow-up till 2019 (8). Considering the similar genetic and cultural factors shared by the East Asian population, the lower burden of NAFLD in our study could be explained by the other important factors, such as health education and counseling. We previously performed the “CCI-HBV demonstration areas” project for 10 years in Zhejiang, which provided well-directed health education that improved patients' self-management of HBV infection and their compliance and understanding to disease prevention and treatment (12). In addition, the low-salt and low-fat dietary habits in Zhejiang areas might also play roles here since NAFLD was found closely associated with high serum lipid levels and obesity. Thus, providing health education counseling and improving unhealthy living habits were warranted in high-prevalence NAFLD areas to reduce the disease burden.

Our study also illustrated the relation between NAFLD and the presence of metabolic factors and underlying diseases. We identified that waist circumference, BMI, TG, LDL, and DM influence factors, which is well-aligned with previous studies. A large-scale case-control study from Central China investigated 7,371 people revealed that the presence of NAFLD was closely linked with a metabolic index, including high TC, high TG, high uric acid, and low HDL, in the elderly (18). Another review article focusing on adolescents and young adults concluded that obesity, DM, total body fat, and other unhealthy lifestyle habits (e.g., smoking, alcohol) were the main risk factors of NAFLD (19). This evidence suggested that patients with metabolic problems (exampled by central obesity, high hypertriglyceridemia, high BMI, and underlying DM) were more prone to develop fatty liver disease. Precisely for this reason, a new term, “Metabolic-associated fatty liver disease,” was recently proposed considering the metabolic pathogenesis in fatty liver (20), which warranted healthcare providers to pay more attention to systemic metabolic conditions instead of local issues confined with the liver in clinical practice.

We further did a subgroup analysis according to DM status. We indicated that HBeAg-negative hepatitis and immunotolerant have a significantly lower prevalence of NAFLD than past infections in non-diabetic patients. However, such association was not suggested in the diabetic subgroup, possibly due to the limited sample size. Multiple research studies have described the association between HBV-related viral factors and NAFLD. Several clinical studies indicated that NAFLD was inversely associated with CHB in both Asian and Caucasian populations, consistent with our findings (4, 8, 21). Especially, Huang et al. pointed that the current infection of CHB groups had a lower prevalence than past infection (4). However, the mechanistic role of HBV infection in NAFLD pathogenesis remained unclear (22). Some researchers attributed the association to adiponectin, which exhibited anti-inflammatory roles and could alleviate steatosis function (23). NAFLD patients tended to have a decreased serum adiponectin level, while HBV is reported to have a potential role in upregulated adiponectin *via* viral protein HBx (24, 25). Moreover, HBV might also affect hepatic cholesterol metabolism by increasing LDL receptor expression and 3-hydroxy-3-methylglutharyl-coenzyme A reductase *via* the pre-S1 domain of viral envelope (26, 27). However, some scientists argued that there is no association between HBV type and NAFLD, based on the results of a cohort study and molecular analysis (11), which is not completely resolved in our study (e.g., the association between HBeAg-positive hepatitis, HBeAg-negative hepatitis, immunotolerant), possibly due to limited sample size. Thus, a large-scale population study is necessary to address this unsettled association between various HBV infection types and NAFLD.

The strengths of this study include its relatively large-scale community-based population in Southeast China, detailed prevalence and incidence descriptive analysis, and comprehensive observation of an association between various metabolic indexes, underlying diseases, viral factors, and NAFLD. However, there are has some limitations in this study, and results should be interpreted with caution. First, the epidemiology of NAFLD in the CHB population had been already examined by several studies previously. Yet, in our research, we simultaneously illustrate comprehensive associations between viral, metabolic, and underlying disease factors. Second, the recruitment of cohort was in 2012 while ultrasound-based follow-up was introduced in 2017, the actual follow-up time for NAFLD was shortened in comparison with the whole natural cohort. However, we will keep following this cohort and report updated findings in the future. Third, our results didn't indicate any new findings of the metabolic features associated with NAFLD, as well as the different stages of NAFLD. Fourth, there was no available data on antiviral therapy, liver fibrosis tested by Fibroscan, or “gold standard” diagnosis of liver biopsy, which might not reveal any other potential associations from drug fibrosis and underestimate the actual prevalence and incidence. Fifth, the sample size was relatively small, which might undermine the evaluation of the possible association between NAFLD and HBV serological status. Sixth, the median age of the study population was 63.0 years old in 2019, which would possibly make our conclusion less persuasive for the younger people.

## Conclusion

In conclusion, we reported a lower NAFLD prevalence and incidence rate than other East Asian studies. Considering our previously performed “CCI-HBV demonstration areas” work, providing health education counseling and improving unhealthy living habits were extremely important in reducing the NAFLD burden. Metabolic factors and DM were identified as associated with NAFLD, which endorsed the metabolic pathogenesis in fatty liver development. Viral factors were also connected with NAFLD in the non-diabetic subgroup, the prevalence of NAFLD was lower in HBeAg-negative hepatitis and immunotolerant type than in the past infection type. However, further research studies that focus on revealing the mechanism and include a more significant number of populations are still required in the future to explain the impact of various HBV infection types.

## Data Availability Statement

The raw data supporting the conclusions of this article will be made available by the authors, without undue reservation.

## Ethics Statement

The studies involving human participants were reviewed and approved by Ethics Committee of the First Affiliated Hospital, College of Medicine, Zhejiang University. The patients/participants provided their written informed consent to participate in this study.

## Author Contributions

LL, YZ, and KX designed the study. YZ, HH, and WW collected the data. YZ analyzed the data, interpreted the results, and wrote the manuscript. KX and MD revised the manuscript from the preliminary draft to submission. LL supervised the whole study. All authors contributed to the article and approved the submitted version.

## Conflict of Interest

The authors declare that the research was conducted in the absence of any commercial or financial relationships that could be construed as a potential conflict of interest.

## Abbreviations

NAFLD: non-alcohol fatty liver disease
CHB: chronic hepatitis B
HBV: hepatitis B virus
HBeAg/HBeAb: hepatitis B e antigen/antibody
BMI: body mass index
TG: triglyceride
LDL: low-density lipoprotein
TC: total cholesterol
ALP: alkaline phosphatase
DM: diabetes mellitus.
