# Supplementary material for: Prevalence and Incidence of Non-alcohol Fatty Liver Disease in Chronic Hepatitis B Population in Southeast China: A Community-Based Study
Source: Front Med (Lausanne). 2021 Jul 19;8:683872. doi: 10.3389/fmed.2021.683872 (PMC8326512; doi:10.3389/fmed.2021.683872)
Supplement: Supplementary file 1 [file Data_Sheet_1.docx]

**Prevalence and incidence of non-alcohol fatty liver disease in chronic hepatitis B population in Southeast China: a community-based study**

**Supplementary material**

Supplementary S1. Categorization of continuous variables.

Supplementary S2. HBV type classification according to HBV serology, HBV DNA, and liver function.

Supplementary Table 1. Characteristics of chronic HBV patients in 2019 (last visit of follow up).

Supplementary Table 2. Characteristics of diabetic and non-diabetic subgroup in 2019 (last visit of follow up)

Supplementary Table 3. Characteristics of chronic HBV patients with negative results of baseline abdominal ultrasound in 2017 (baseline)

Supplementary Table 4. Characteristics of diabetic and non-diabetic subgroup with negative results of baseline abdominal ultrasound in 2017 (baseline)

**Supplementary S1.** **Categorization of continuous variables**

| **Continuous variables** | **Categorization** |
| --- | --- |
| **Waist Circumference (cm)** |  |
| Normal | Male<85, Female<80 |
| Obese | Male≥85, Female≥80 |
| **BMI** |  |
| Normal | <24 |
| Overweight | 24-28 |
| Obese | >28 |
| **TG (mmol/L)** |  |
| Optimal | <1.7 |
| Intermediate | 1.7-2.25 |
| High | >2.25 |
| **LDL (mmol/L)** |  |
| Optimal | <3.36 |
| Intermediate | 3.36-4.11 |
| High | >4.11 |

**Reference:**

1. Gao M, Wei Y X, Lyu J, et al. [The cut-off points of body mass index and waist circumference for predicting metabolic risk factors in Chinese adults] (in Chinese) [J]. Chinese Journal of Epidemiology= Zhonghua liuxingbingxue zazhi, 2019, 40(12): 1533-1540.

2. Zhu J R, Gao R L, Zhao S P, et al. [Guidelines for the prevention and treatment of dyslipidemia in adults in China (2016 revised edition)] (in Chinese) [J]. Chinese Circulation Journal, 2016, 31(10):937-950.

**Supplementary S2. HBV type classification according to HBV serology, HBV DNA, and liver function**

Immunotolerant: HBeAg positive, HBV DNA positive, typically > 10^6^ IU/ml, normal ALT and/or AST.

Inactive carrier: HBeAg negative, HBV DNA negative, < 10^3^ IU/ml, normal ALT and/or AST.

HBeAg positive hepatitis: HBeAg positive, HBV DNA positive, persistently or intermittently elevated ALT and/or AST.

HBeAg negative hepatitis (HBeAg negative, HBV DNA positive, persistently or intermittently elevated ALT and/or AST.

Past HBV infection: positive anti-HBc without HBsAg in HBV serology.

**Reference:**

1. Chinese Medical Association. [The guidelines of prevention and treatment for chronic hepatitis B (2019 version)] (in Chinese) [J]. Zhonghua gan zang bing za zhi= Zhonghua ganzangbing zazhi= Chinese journal of hepatology, 2019, 27(12): 938-961

2. Terrault N A, Lok A S F, McMahon B J, et al. Update on prevention, diagnosis, and treatment of chronic hepatitis B: AASLD 2018 hepatitis B guidance[J]. Hepatology, 2018, 67(4): 1560-1599.

**Supplementary Table 1.** **Characteristics of chronic HBV patients in 2019 (last visit of follow up)**

|  | **NAFLD (n=247)** | **Non-NAFLD (n=676)** | **Total (n=923)** | ***p*** |
| --- | --- | --- | --- | --- |
| **Age** | 63.0 (56.0-69.0) | 64.0 (55.0-70.0) | 63.0 (56.0-70.0) | 0.827 |
| **Gender** |  |  |  | 0.350 |
| male | 112 (45.3%) | 330 (48.8%) | 442 (47.9%) |  |
| female | 135 (54.7%) | 346 (51.2%) | 481 (52.1%) |  |
| **HBV type** |  |  |  | 0.131 |
| immunotolerant | 7 (2.8%) | 48 (7.1%) | 55 (6.0%) |  |
| inactive carrier | 199 (80.6%) | 523 (77.4%) | 722 (78.2%) |  |
| e+ hepatitis | 6 (2.4%) | 20 (3.0%) | 26 (2.8%) |  |
| e- hepatitis | 21 (8.5%) | 58 (8.6%) | 79 (8.6%) |  |
| past infection | 14 (5.7%) | 27 (4.0%) | 41 (4.4%) |  |
| **HBV DNA, log 10** | 3.00 (3.00-3.54) | 3.02 (3.00-4.11) | 3.00 (3.00-3.97) | 0.004 |
| **Liver function** |  |  |  |  |
| ALT, U/L | 26.0 (20.0-40.8) | 21.0 (16.2-30.0) | 22.3 (17.0-33.0) | <0.001 |
| AST, U/L | 25.0 (21.0-32.0) | 25.0 (21.0-31.0) | 25.0 (21.0-31.0) | 0.392 |
| Bilirubin, umol/L | 14.5 (11.7-18.5) | 15.1 (11.8-19.1) | 14.9 (11.7-18.9) | 0.001 |
| AKP, U/L | 97.0 (81.0-117.0) | 98.0 (78.0-120.0) | 97.0 (79.0-119.0) | 0.341 |
| γGT, U/L | 24.0 (18.0-34.0) | 19.0 (15.0-29.0) | 21.0 (15.0-119.0) | 0.835 |
| albumin, g/L | 47.55±3.05 | 47.03±2.99 | 47.17±3.01 | <0.001 |
| **Renal function** | |  |  |  |
| Creatinine, umol/L | 66.0 (56.0-76.0) | 66.3 (58.6-76.0) | 66.3 (58.0-76.0) | 0.389 |
| BUN, mmol/L | 5.09 (4.28-6.06) | 5.47 (4.50-6.45) | 5.36 (4.44-6.36) | 0.009 |
| **alpha fetoprotein** |  |  |  | 0.063 |
| - | 245 (99.2%) | 671 (99.3%) | 916 (99.2%) | 0.913 |
| + | 2 (0.8%) | 5 (0.7%) | 7 (0.8%) |  |
| **Body index** |  |  |  |  |
| BMI | 26.73±3.01 | 23.37±2.93 | 24.27±3.30 |  |
| Waist, cm | 90.0 (84.0-95.0) | 81.0 (75.0-87.0) | 84.0 (77.0-90.0) | <0.001 |
| **Blood Pressure (n=836)** | |  |  |  |
| Systolic, mmHg | 143 (133-156) | 136 (125-151) | 140 (127-153) |  |
| Diastolic, mmHg | 84 (77-91.8) | 81 (74-89) | 82 (75-90) | <0.001 |
| **Lipid** |  |  |  | 0.001 |
| TC, mmol/L | 5.04±0.97 | 4.63±0.90 | 4.75±0.93 |  |
| LDL, mmol/L | 2.73 (2.23-3.36) | 2.38 (1.98-2.82) | 2.47 (2.02-2.97) | <0.001 |
| HDL, mmol/L | 1.34 (1.16-1.58) | 1.46 (1.25-1.70) | 1.43 (1.21-1.65) | <0.001 |
| TG, mmol/L | 1.85 (1.19-2.50) | 1.03 (0.77-1.48) | 1.15 (0.82-1.85) | <0.001 |
| **Diabetes mellitus** |  |  |  | <0.001 |
| - | 220 (89.1%) | 648 (95.9%) | 868 (94.0%) | <0.001 |
| + | 27 (10.9%) | 28 (4.1%) | 55 (6.0%) |  |

Data are median (IQR) or median±S.D for continuous variables, n (%) for categorical variables.

P values are calculated using t-test or Mann-Whitney U test for continuous variables and chi-square test or Fisher’s exact test for categorical variables.

ALT: alanine transaminase; AST: aspartate transaminase; ALP: alkaline phosphatase; γGT: gamma-glutamyl transferase; BUN: blood urine nitrogen; BMI: body mass index; TC: total cholesterol; LDL: low density lipoprotein; HDL: high density lipoprotein; TG: triglycerides.

**Supplementary Table 2.** **Characteristics of diabetic and non-diabetic subgroup in 2019 (last visit of follow up)**

|  | **non-DM (n=868)** | **DM (n=55)** | **Total (n=923)** | **p** |
| --- | --- | --- | --- | --- |
| **Age** | 63.0 (55.0-70.0) | 64.0 (60.0-69.0) | 63.0 (56.0-70.0) | 0.192 |
| **Gender** |  |  |  | 0.137 |
| Male | 421 (48.5%) | 21 (38.2%) | 442 (47.9%) |  |
| Female | 447 (51.5%) | 34 (61.8%) | 481 (52.1%) |  |
| **AST, U/L** | 28.0 (23.0-35.0) | 27.0 (22.0-35.0) | 28.0 (23.0-35.0) | 0.374 |
| <40 | 709 (81.7%) | 48 (87.3%) | 757 (82.0%) | 0.295 |
| ≥40 | 159 (18.3%) | 7 (12.7%) | 166 (18.0%) |  |
| **Waist, cm** | 84.00 (77.00-90.00) | 86.00 (78.00-93.00) | 84.0 (77.0-90.0) | 0.867 |
| **BMI** | 24.16 (21.97-26.31) | 25.48 (22.21-27.38) | 24.17 (22.01-26.43) | 0.481 |
| **TG, mmol/L** | 1.15 (0.82-1.83) | 1.33 (0.80-2.09) | 1.15 (0.82-1.85) | 0.020 |
| **TC, mmol/L** | 4.71 (4.08-5.37) | 4.83 (4.05-5.56) | 4.71 (4.08-5.37) | 0.035 |
| **LDL, mmol/L** | 2.47 (2.02-2.96) | 2.50 (2.02-3.10) | 2.47 (2.02-2.97) | 0.333 |
| **HDL, mmol/L** | 1.43 (1.21-1.65) | 1.42 (1.19-1.61) | 1.43 (1.21-1.65) | 0.344 |
| **HBV Type** |  |  |  | 0.034 |
| past infection | 40 (4.6%) | 1 (1.8%) | 41 (4.4%) |  |
| e- hepatitis | 76 (8.8%) | 3 (5.5%) | 79 (8.6%) |  |
| e+ hepatitis | 23 (2.6%) | 3 (5.5%) | 26 (2.8%) |  |
| inactive carrier | 674 (77.6%) | 48 (87.3%) | 722 (78.2%) |  |
| immunotolerant | 55 (6.3%) | 0 (0%) | 55 (6.0%) |  |

Data are median (IQR) or median±S.D for continuous variables, n (%) for categorical variables.

p values are calculated using t-test or Mann-Whitney U test for continuous variables and chi-square test or Fisher’s exact test for categorical variables.

DM: diabetes mellitus; AST: aspartate transaminase; BMI: body mass index; TG: triglycerides; TC: total cholesterol; LDL: low density lipoprotein; HDL: high density lipoprotein; HBV: hepatitis B virus.

**Supplementary Table 3. Characteristics of chronic HBV patients with negative results of baseline abdominal ultrasound in 2017 (baseline)**

|  | **Incidence (n=37)** | **Negative(n=676)** | **Total (n=713)** | ***p*** |
| --- | --- | --- | --- | --- |
| **Age (in 2017)** | 61.0 (55.5-67.0) | 62.0 (53.0-68.0) | 62.0 (53.0-68.0) | 0.455 |
| **Gender** |  |  |  | 0.734 |
| male | 17 (45.9%) | 330 (48.8%) | 347 (48.7%) |  |
| female | 20 (54.1%) | 346 (51.2%) | 366 (51.3%) |  |
| **HBV type** | |  |  | 0.036 |
| immunotolerant | 0 (0.0%) | 48 (7.1%) | 48 (6.7%) |  |
| inactive carrier | 31 (83.8%) | 523 (77.4%) | 554 (77.7%) |  |
| e+ hepatitis | 3 (8.1%) | 20 (3.0%) | 23 (3.2%) |  |
| e- hepatitis | 0 (0.0%) | 58 (8.6%) | 58 (8.1%) |  |
| past infection | 3 (8.1%) | 27 (4.0%) | 30 (4.2%) |  |
| **HBV DNA, log** | 3.01 (3.00-3.85) | 3.02 (3.00-4.11) | 3.02 (3.00-4.08) | 0.866 |
| **Liver function** |  |  |  |  |
| ALT, U/L | 24.0 (18.5-32.5) | 21.0 (16.3-30.0) | 21.2 (16.5-30.0) | 0.062 |
| ≥40 | 7 (6.8%) | 30 (4.9%) | 37 (5.2%) | 0.427 |
| AST, U/L | 24.0 (20.2-29.0) | 25.0 (21.0-31.5) | 25.0 (21.0-31.0) | 0.383 |
| Bilirubin, umol/L | 16.4 (13.8-21.3) | 15.6 (12.3-19.5) | 15.7 (12.4-19.6) | 0.154 |
| ALP, U/L | 89.0 (73.5-107.5) | 98.0 (78.0-120.0) | 97.0 (78.0-119.0) | 0.136 |
| ≥125 | 4 (10.8%) | 140 (20.7%) | 144 (20.2%) | 0.144 |
| γGT, U/L | 24.0 (19.0-27.5) | 19.0 (15.0-29.0) | 20.0 (15.0-29.0) | 0.036 |
| ≥50 | 4 (5.4%) | 33 (5.2%) | 37 (5.2%) | 0.929 |
| albumin, g/L | 46.4 (44.1-47.4) | 45.5 (43.7-46.9) | 45.6 (43.7-47.0) | 0.191 |
| **Renal function (n=635)** | |  |  |  |
| Creatinine, umol/L | 67.1 (59.0-77.4) | 66.3 (58.6-76.0) | 66.3 (58.9-76.0) | 0.732 |
| BUN, mmol/L | 5.34 (4.38-6.23) | 5.47 (4.50-6.45) | 5.46 (4.50-6.43) | 0.605 |
| **alpha fetoprotein** | |  |  | 0.203 |
| - | 36 (97.3%) | 671 (99.3%) | 707 (99.2%) |  |
| + | 1 (2.7%) | 5 (0.7%) | 6 (0.8%) |  |
| **Body index** | |  |  |  |
| BMI | 25.56±2.46 | 23.40±2.96 | 23.51±2.97 | <0.001 |
| Waist, cm | 88.0 (83.0-93.5) | 81.0 (75.0-87.0) | 81.0 (76.0-87.0) | <0.001 |
| **Blood Pressure (n=656)** | |  |  |  |
| Systolic, mmHg | 141 (133-154) | 136 (125-151) | 137 (126-152) | 0.038 |
| Diastolic, mmHg | 84 (75-90) | 81 (74-89) | 82 (74-89) | 0.249 |
| **Lipid** |  |  |  |  |
| TC, mmol/L | 5.06±1.18 | 4.63±0.91 | 4.66±0.93 | 0.001 |
| LDL, mmol/L | 2.94 (2.10-3.43) | 2.38 (1.98-2.82) | 2.40 (1.99-2.85) | <0.001 |
| HDL, mmol/L | 1.43 (1.19-1.68) | 1.46 (1.25-1.70) | 1.46 (1.24-1.70) | 0.454 |
| TG, mmol/L | 1.70 (1.11-2.12) | 1.03 (0.77-1.48) | 1.05 (0.78-1.56) | <0.001 |
| **Diabetes mellitus** |  |  |  | 0.078 |
| - | 33 (89.2%) | 648 (95.9%) | 681 (95.5%) |  |
| + | 4 (10.8%) | 28 (4.1%) | 32 (4.5%) |  |

Data are median (IQR) or median±S.D for continuous variables, n (%) for categorical variables.

P values are calculated using t-test or Mann-Whitney U test for continuous variables and chi-square test or Fisher’s exact test for categorical variables.

ALT: alanine transaminase; AST: aspartate transaminase; ALP: alkaline phosphatase; γGT: gamma-glutamyl transferase; BUN: blood urine nitrogen; BMI: body mass index; TC: total cholesterol; LDL: low density lipoprotein; HDL: high density lipoprotein; TG: triglycerides.

**Supplementary Table 4. Characteristics of diabetic and non-diabetic subgroup with negative results of baseline abdominal ultrasound in 2017 (baseline)**

|  | **non-DM (n=681)** | **DM (n=32)** | **Total (n=713)** | ***p*** |
| --- | --- | --- | --- | --- |
| **Age group** | 63.0 (55.0-70.0) | 64.0 (60.3-69.0) | 64.0 (55.0-70.0) | 0.335 |
| ≤60 | 269 (39.5%) | 8 (25.0%) | 277 (38.8%) | 0.100 |
| 61- | 412 (60.5%) | 24 (75.0%) | 436 (61.2%) |  |
| **Gender** |  |  |  | 0.836 |
| Male | 332 (48.8%) | 15 (46.9%) | 347 (48.7%) |  |
| Female | 349 (51.2%) | 17 (53.1%) | 366 (51.3%) |  |
| **ALP, U/L** | 97.0 (78.0-119.0) | 102.5 (76.8-138.5) | 97.0 (78.0-119.0) | 0.461 |
| <125 | 545 (80.0%) | 24 (75.0%) | 569 (79.8%) | 0.489 |
| ≥125 | 136 (20.0%) | 8 (25.0%) | 144 (20.2%) |  |
| **Waist** | 81.0 (75.0-87.0) | 82.5 (76.3-90.0) | 81.0 (76.0-87.0) | 0.123 |
| **BMI** | 23.32 (21.45-25.25) | 24.05 (21.52-25.82) | 23.39 (21.47-25.39) | 0.265 |
| normal (<24) | 362 (56.6%) | 16 (50.0%) | 378 (56.3%) | 0.359 |
| overweight (24-27) | 198 (30.9%) | 9 (28.1%) | 207 (30.8%) |  |
| obese (27-) | 80 (12.5%) | 7 (21.9%) | 87 (12.9%) |  |
| **TG** | 1.06 (0.78-1.56) | 1.01 (0.75-1.73) | 1.05 (0.78-1.56) | 0.983 |
| optimal (<1.7) | 470 (78.9%) | 23 (71.9%) | 493 (78.5%) | 0.626 |
| intermediate (1.7-2.25) | 77 (12.9%) | 6 (18.8%) | 83 (13.2%) |  |
| high (>2.25) | 49 (8.2%) | 3 (9.4%) | 52 (8.3%) |  |
| **TC** | 4.63 (4.05-5.30) | 4.57 (3.77-5.46) | 4.63 (4.04-5.30) | 0.807 |
| **LDL** | 2.40 (1.98-2.84) | 2.40 (2.00-3.01) | 2.40 (1.99-2.85) | 0.719 |
| optimal (<3.36) | 544 (90.5%) | 26 (81.3%) | 570 (90.0%) | 0.288 |
| intermediate (3.36-4.11) | 50 (8.3%) | 5 (15.6%) | 55 (8.7%) |  |
| high (>4.11) | 7 (1.2%) | 1 (3.1%) | 8 (1.3%) |  |
| **HDL** | 1.46 (1.25-1.70) | 1.43 (1.19-1.68) | 1.46 (1.24-1.70) | 0.532 |
| **HBV Type** |  |  |  | 0.118 |
| past infection | 29 (4.3%) | 1 (3.1%) | 30 (4.2%) |  |
| e- hepatitis | 56 (8.2%) | 2 (6.3%) | 58 (8.1%) |  |
| e+ hepatitis | 20 (2.9%) | 3 (9.4%) | 23 (3.2%) |  |
| inactive carrier | 528 (77.5%) | 26 (81.3%) | 554 (77.7%) |  |
| immunotolerant | 48 (7.0%) | 0 (0%) | 48 (6.7%) |  |

Data are median (IQR) or median±S.D for continuous variables, n (%) for categorical variables.

P values are calculated using t-test or Mann-Whitney U test for continuous variables and chi-square test or Fisher’s exact test for categorical variables.

DM: diabetes mellitus; AST: aspartate transaminase; ALP: alkaline phosphatase; BMI: body mass index; TG: triglycerides; TC: total cholesterol; LDL: low density lipoprotein; HDL: high density lipoprotein; HBV: hepatitis B virus.
